# Registration accuracy for MR images of the prostate using a subvolume based registration protocol

**DOI:** 10.1186/1748-717X-6-73

**Published:** 2011-06-16

**Authors:** Joakim H Jonsson, Patrik Brynolfsson, Anders Garpebring, Mikael Karlsson, Karin Söderström, Tufve Nyholm

**Affiliations:** 1 Radiation Physics, Department of Radiation Sciences, Umeå University, 90187 Umeå, Sweden; 2 Oncology, Department of Radiation Sciences, Umeå University, 90187 Umeå, Sweden

**Keywords:** MRI, image registration, prostate, radiotherapy, subvolume, localized, cancer

## Abstract

**Background:**

In recent years, there has been a considerable research effort concerning the integration of magnetic resonance imaging (MRI) into the external radiotherapy workflow motivated by the superior soft tissue contrast as compared to computed tomography. Image registration is a necessary step in many applications, e.g. in patient positioning and therapy response assessment with repeated imaging. In this study, we investigate the dependence between the registration accuracy and the size of the registration volume for a subvolume based rigid registration protocol for MR images of the prostate.

**Methods:**

Ten patients were imaged four times each over the course of radiotherapy treatment using a T2 weighted sequence. The images were registered to each other using a mean square distance metric and a step gradient optimizer for registration volumes of different sizes. The precision of the registrations was evaluated using the center of mass distance between the manually defined prostates in the registered images. The optimal size of the registration volume was determined by minimizing the standard deviation of these distances.

**Results:**

We found that prostate position was most uncertain in the anterior-posterior (AP) direction using traditional full volume registration. The improvement in standard deviation of the mean center of mass distance between the prostate volumes using a registration volume optimized to the prostate was 3.9 mm (p < 0.001) in the AP direction. The optimum registration volume size was 0 mm margin added to the prostate gland as outlined in the first image series.

**Conclusions:**

Repeated MR imaging of the prostate for therapy set-up or therapy assessment will both require high precision tissue registration. With a subvolume based registration the prostate registration uncertainty can be reduced down to the order of 1 mm (1 SD) compared to several millimeters for registration based on the whole pelvis.

## Introduction

The role of magnetic resonance imaging (MRI) in modern prostate external radiotherapy treatments has in recent years attracted a lot of scientific attention. The applications span from MRI based treatment planning [1-4] to assessment of treatment response using different MRI techniques such as dynamic contrast enhanced MRI (DCE-MRI) [5,6], diffusion weighted imaging (DWI) [7,8] and magnetic resonance spectroscopy (MRS) [9]. It is widely accepted in the radiotherapy community that MRI is the preferred choice for target delineation of e.g. prostate, due to its superior soft tissue contrast [10]. It has also been shown that multi-modal registration between MRI and computed tomography (CT) increases the systematic uncertainty of the treatment [11]. It is therefore desirable to develop an MR only workflow where the treatment planning, patient positioning and treatment response evaluation is based on MR imaging. The soft tissue contrast and non-ionizing properties of the MRI scanner make it ideal for daily patient positioning. Several solutions on integration of MRI into the external radiotherapy procedure for this purpose have been suggested in literature, e.g. integrated MR scanner-accelerator solutions [12,13] or using a patient transport solution from a nearby MR scanner [14].

Image registration is an essential part of medical image analysis. It can be used to combine multi-modal images via image fusion [15,16], align four dimensional images [17], correct for patient setup errors [18], respiratory tracking [19], automatic image segmentation [20], contour propagation [21] and many other purposes. All of these applications are present in a modern radiotherapy department during treatment planning, the treatment delivery as well as during patient follow-up and tumor response evaluation.

In patients with clinically localized prostate cancer, traditional rigid registration between image volumes acquired at different times may not perform adequately with respect to the tumor shape and position, since the prostate can move with respect to the bony anatomy and external patient contour [22]. This makes ordinary rigid registration, based on the entire patient anatomy, imprecise. In order to align the prostate volume with high precision, there is a need for a registration of the prostate only. One way of accomplishing this is the use of intra-prostatic fiducial markers. The radio opaque markers are implanted into the prostate gland, and can thereafter be visualized using most imaging modalities. By manually defining the markers in the two image sets, the images can be registered so that the markers are as close to each other as possible. This implicitly registers the images with focus on the prostate area, provided that the markers have not migrated within the prostate gland.

A non-invasive path to localized registration of mobile organs is subvolume based rigid registration taking only the volume of interest into account. For patient positioning, the subvolume based rigid registration approach has the advantage that the registration results can be readily interpreted as couch movements, making instant adjustment of patient position possible. The properties of subvolume based registration have been investigated for repeat CT [23] and cone beam CT (CBCT) [24], but to our knowledge not yet for MRI.

In the present study we investigate the precision of subvolume based rigid registration of the prostate for ten patients with four repeat MR scans each. The aim was to quantify the registration precision and its dependence of the registration volume for a mean square metric based algorithm, i.e. determine the optimal size of the registration volume to be used for alignment of MR images for treatment response evaluation and external radiotherapy purposes.

## Methods

### Patients

Ten patients with median age 58 years (range 52-69 years) scheduled for pre-treatment pelvic MRI scans were included in the study. All patients were treated with fractionated external radiotherapy using three different protocols. The choice of radiotherapy protocol did not influence the prostate delineation to be used in the study.

### Imaging

Prior to treatment the patients were imaged with an Espree 1.5 T MR scanner (Siemens Medical, Erlangen, Germany) using a T2 weighted high resolution 3D sequence (SPACE) with axial slices (repetition time was 1500 ms, echo time was 209 ms, number of slice averages was 1, slice thickness 1.7 mm, 120 slices, pixel bandwidth 590 Hz/pixel, flip angle 150 degrees, matrix size 384 × 348, in-plane pixel size 1.17 × 1.17 mm). This MR sequence is part of the normal clinical protocol and is used for target definition. The same MR sequence was repeated three times during the treatment duration, yielding a total of four MR image sets for each patient. The patients were placed on a flat tabletop insert during the MR imaging, and the images were acquired with the body matrix and spine coil.

During the MR imaging, the patients were placed in the scanner in supine position with the standard treatment fixation devices, which consist of a knee cushion that prevents rotation of the pelvis.

### Delineation

**Figure 1:**
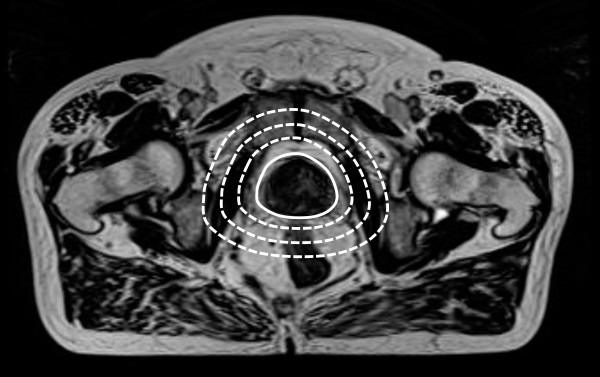
**Registration volumes**. The figure demonstrates an MR image with the different registration volumes RV_0 _(solid line), RV_1_, RV_2 _and RV_3 _(dotted lines).

The prostate gland registration volume, defined as RV_0_, was delineated by a hospital physicist in collaboration with a radio oncologist on the pre-treatment image sets. RV_0 _included the entire prostate gland excluding the seminal vesicles. 3D margins of 1, 2 and 3 cm were added to RV_0 _to create different registration volumes denoted as RV_1_, R2_V _and RV_3_, see Figure 1. A volume corresponding to RV_0 _was delineated on the treatment image sets. This volume did not affect the registration in any way, but was used solely for analysis purposes.

### Registration

In order to register the images with respect to the soft tissue in the target and not take the bony anatomy and external patient contour into account, the metric calculation needs to be constructed in such a way that only values within a specific region of interest, i.e. the registration volume, are taken into account. This was accomplished by use of binary volumes, i.e. masks, which define in what region the metric values should be calculated. These masks were created by converting the contours delineated by the authors to binary volumes.

We used MATLAB (MathWorks, Natick, MA) and the Insight Toolkit (ITK) to develop a method for MR-MR image registration. Since it was a single modality registration problem, we used a mean square metric. A step gradient descent approach, the VersorTransformOptimizer, was used for the optimization.

We registered the pre-treatment MRI to the other 3 image sets for each patient, using the complete volume, RV_0 _mask, RV_1 _mask, RV_2 _mask and RV_3 _mask. This yielded a total number of 150 MR-MR registrations.

### Analysis

We quantified the registration uncertainty as the standard deviation of the center of mass distance between the prostate gland (RV_0_) binary masks for each pair of registered images. This measure has a clinical relevance as the center of mass distance vector corresponds to the couch shift vector when positioning the patient. The registration uncertainty was scored for each main direction x (right-left), y (anterior-posterior) and z (cranio-caudal) and for the norm of this vector. We used F-tests to test for significance in the difference of variance in registrations between different pairs of registration volumes.

## Results

**Figure 2:**
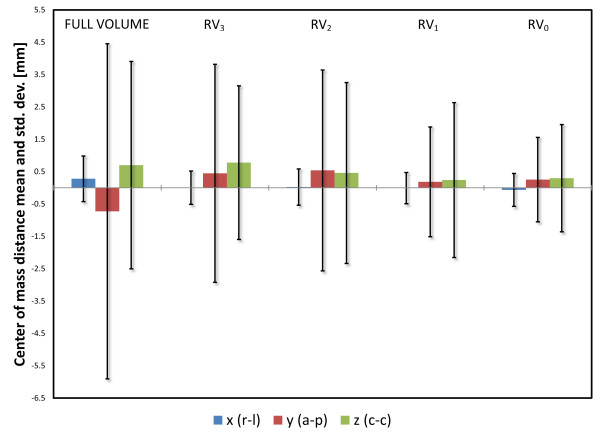
**Registration results**. Center of mass standard deviations per coordinate, grouped by registration volume. The colored bar represents the mean center of mass distance and the error bars displays ± 1 standard deviation. The variance in center of mass distance is stable for the right-left direction, but increases with increasing registration volume size for the other directions.

**Figure 3:**
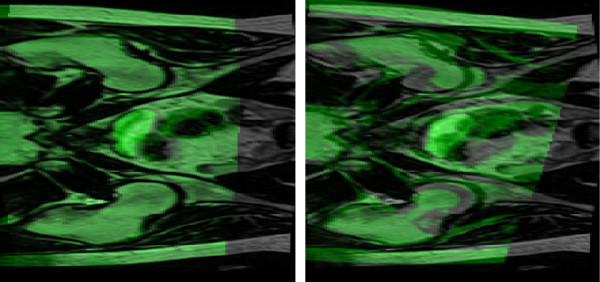
**Failed registration**. The failed registration reflected in the min row in table 1. The fixed image is displayed in grayscale and the moving image is displayed using a green overlay. The full volume registration can be seen to the left and the subvolume based registration using RV_2 _to the right. The misregistration is obvious and is easily detected by visual inspection.

The registrations were performed for all patients and all registration volumes for the MR series, see Figure 2. The standard deviation of the center of mass distance post registration was reduced with a decrease in registration volume. The reduction was most pronounced in the anterior-posterior direction, from 5.2 mm with full volume registration to 1.3 mm (p < 0.001) using RV_0_. In the cranio-caudal direction the standard deviation was reduced from 3.2 mm to 1.7 mm (p < 0.001), and in the right-left direction the reduction of the standard deviation was modest, from 0.7 mm to 0.5 mm (p = 0.08), also using RV_0_. The standard deviation of the norm of the vector was reduced from 2.8 mm to 0.8 mm (p < 0.001). The mean, median and range of the norm improvement are presented in table 1, together with p-values for difference in variance between the specific registration volumes compared to the full volume registrations. Negative numbers indicate that the subvolume based registration failed to produce a better result than the full volume registration. The numbers indicated in the min row all occurred for the same patient image set where registration failed, see Figure 3. Exclusion of this atypical image set would have led to a minimum improvement around -1 mm.

**Table 1:** Registration results

	RV_0_	RV_1_	RV_2_	RV_3_
**Min**	-0.48	-4.39	-9.47	-3.26
**Max**	11.09	11.15	8.76	7.78
**Median**	2.34	1.63	1.32	1.19
**Mean**	3.13	2.40	1.58	1.56
**p**	< 0.001	< 0.001	0.03	0.02

Mean, median and range of improvement (norm) from full volume registration to subvolume based registration with different registration volumes. Negative numbers indicate that the subvolume based registration failed to produce a better result than the full volume registration.

Figure 2 shows that the registration uncertainty in the anterior-posterior direction is more sensitive to the size of the registration volume, compared to the cranio-caudal and right-left directions. For the largest registration volumes RV_2 _and RV_3_, as well as full volume registration, the anterior-posterior direction contributes to the largest part of the total registration uncertainty. This is likely due to the increase in rectal volume included in the registration volume.

The registration volume that gave the most precise results was RV_0 _for 77% of the image pairs, RV_1 _and RV_2 _for 10% of the pairs each, and RV_3 _was most precise only for 3% of the cases. These results are not surprising, since the larger registration volumes include more of the rectum and bladder. Hence, the registration algorithm includes changes in these areas, leading to a degradation of the registration with respect to the prostate.

## Discussion

The results in this study clearly demonstrate that subvolume based rigid registration improves the registration precision within the area of interest. However, as with all registration protocols, there is a need for quality control such as visual inspection to make sure that the registration has not failed. The subvolume based protocol has applications within patient positioning using image guided radiotherapy and when using multiple imaging for treatment response evaluation.

The MR-MR subvolume based registration protocol described in the present study performs optimally when applied to a registration subvolume with no margin added to the prostate gland. In a study by Mclaughlin et al [25] regarding subvolume based registration between MR and CT, the prostate volume with no margin did not result in a successful registration due to the lack of information in the prostate area of the CT. In this study, a 2 cm margin added to the prostate was required to ensure a successful registration.

An alternative approach is non-rigid image registration for treatment adaptation. Chao et al [26] used deformable registration to warp a narrow shell and map contours from a planning CT to CBCT images. Wang et al [27] used deformable registration over the entire volume to map contours from a planning CT to 25 repeat CTs for a prostate patient. A problem with deformable registration for image guided radiotherapy is that it requires online replanning or some other form of plan modification. There is no obvious way to interpret the deformation field into a table movement that can be applied immediately. Instead, the multi leaf collimator must be adapted to the new contour, and the dose distribution should be recalculated. This problem does not occur when using localized rigid registration since the registration transform can be readily interpreted as couch movement to reposition the patient. While online plan modification may increase the accuracy of the delivered dose, it is currently time consuming and not easily implemented in a clinical setting.

The implantation of fiducial gold markers into the prostate for localized rigid registration, while accurate if applied properly, has disadvantages compared to the proposed method of registration; it is invasive and the position of the gold markers in the MR images does not necessarily correspond to the markers actual position, depending on sequence parameters [28]. The proposed method is automatic with no need for user interaction and does not require any additional steps in the workflow. In an external radiotherapy workflow, the registration volume can simply be set to the prostate volume defined by the radio oncologist during target definition.

The resulting uncertainties from this study indicate that a standard deviation of approximately 1 mm can be achieved in an automatic procedure. Data from the CT-based study [23] indicate similar results, based on more registrations but with outlier removal, which was not performed in the current study.

## Conclusions

The subvolume based rigid registration of MR scans of the prostate improves the precision significantly as compared to full volume registration. Our results indicate that the optimal registration volume is the prostate itself without any additional surrounding tissue. The subvolume based registration procedure can be applied in an image guided radiotherapy protocol and can be used for registration of repeated MR-imaging of the prostate.

## Competing interests

The authors declare that they have no competing interests.

## Authors' contributions

JJ gathered the data, delineated the contours in collaboration with KS, created software needed for the study and drafted the manuscript. PB and AG aided in the creation of the registration software. MK participated in the design and coordination of the study. TN conceived the study and helped draft the manuscript. All authors read and approved the final manuscript.
